# Supplementary figures and images for: Trehalose and α-glucan mediate distinct abiotic stress responses in Pseudomonas aeruginosa
Source: PLoS Genet. 2021 Apr 19;17(4):e1009524. doi: 10.1371/journal.pgen.1009524 (PMC8084333; doi:10.1371/journal.pgen.1009524)

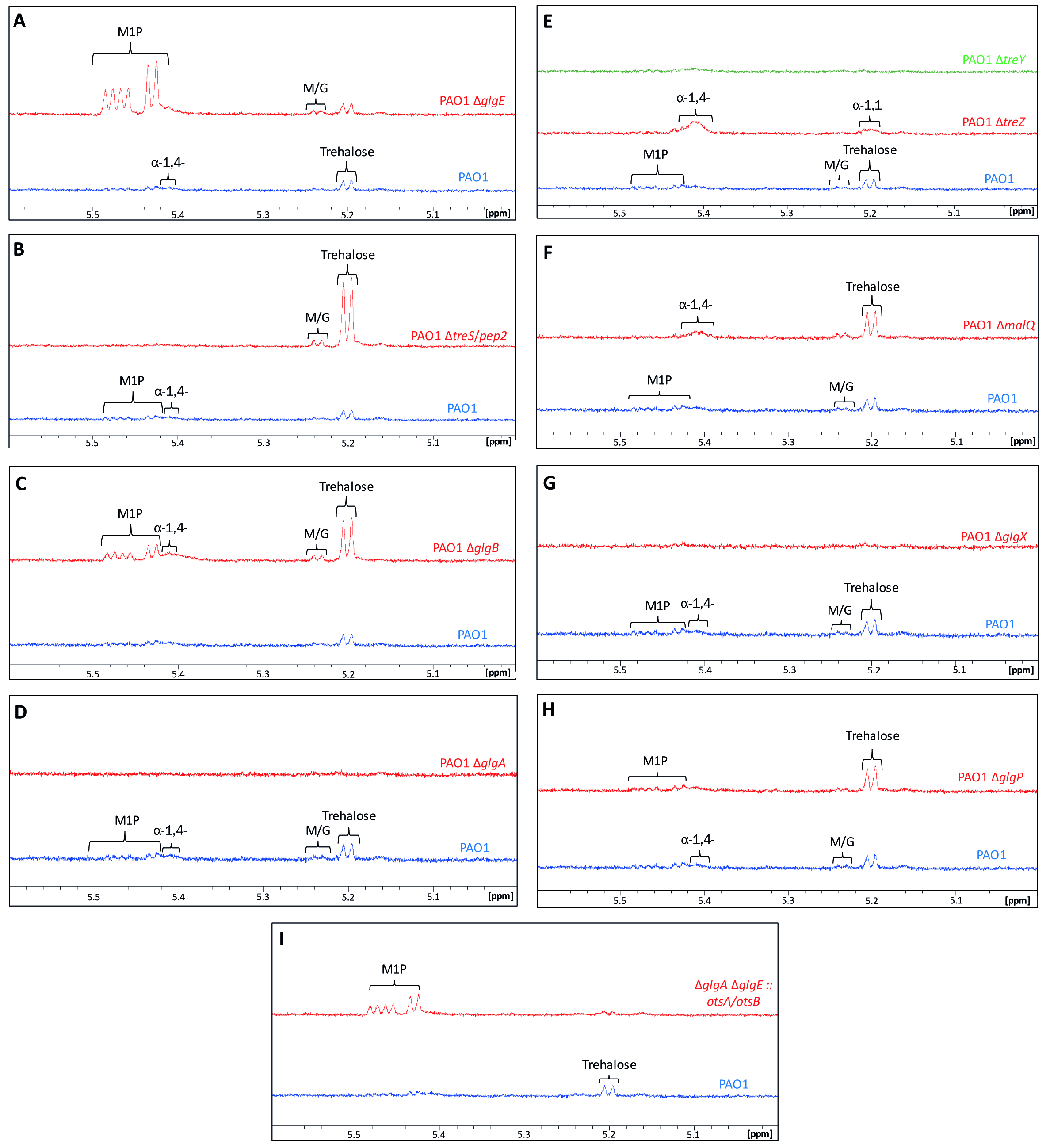

Supplement: S1 Fig — Peaks corresponding to key metabolites: M/G–maltose/glucose, α-1,4-– α-glucan, α-1,1-–probable terminal linkage of maltooligosyltrehalose. Mutant strains are labelled as follows: A) ΔglgE, B) ΔtreS/pep2, C) ΔglgB, D) ΔglgA, E) ΔtreZ/ΔtreY, F) ΔmalQ, G) ΔglgX, H) ΔglgP, I) ΔglgA ΔglgE:: otsA/otsB. (TIF) [file pgen.1009524.s001.tif]

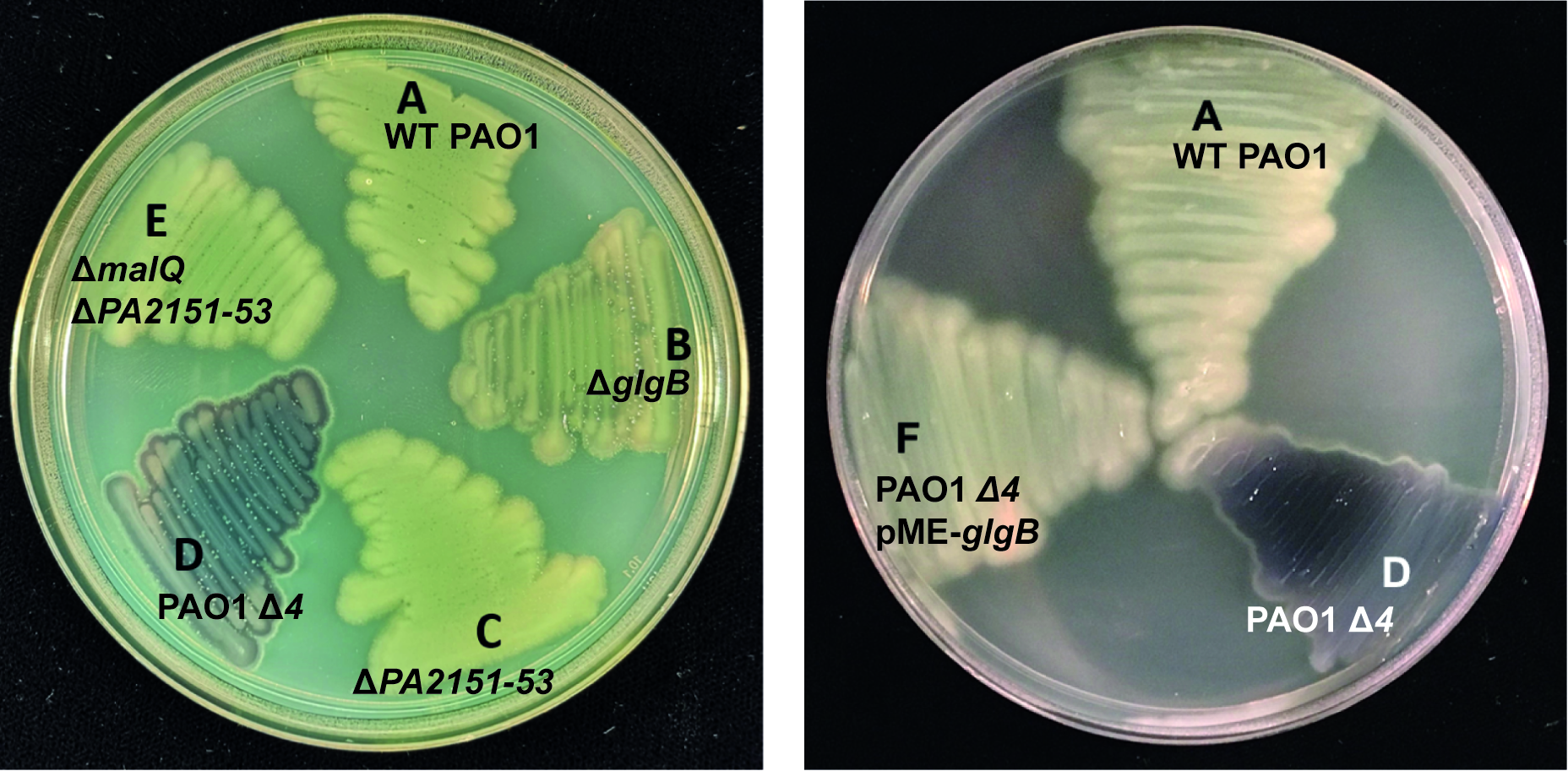

Supplement: S2 Fig — Strains producing insoluble linear α-glucan stain blue-purple. Mutant strains are labelled as follows: A) Wild-type PAO1, B) ΔglgB, C) ΔPA2151-53, D) ΔtreY ΔPA2151-53 (PAO1 Δ4), E) ΔmalQ ΔPA2151-53, F) PAO1 Δ4 pME-glgB. (TIF) [file pgen.1009524.s002.tif]

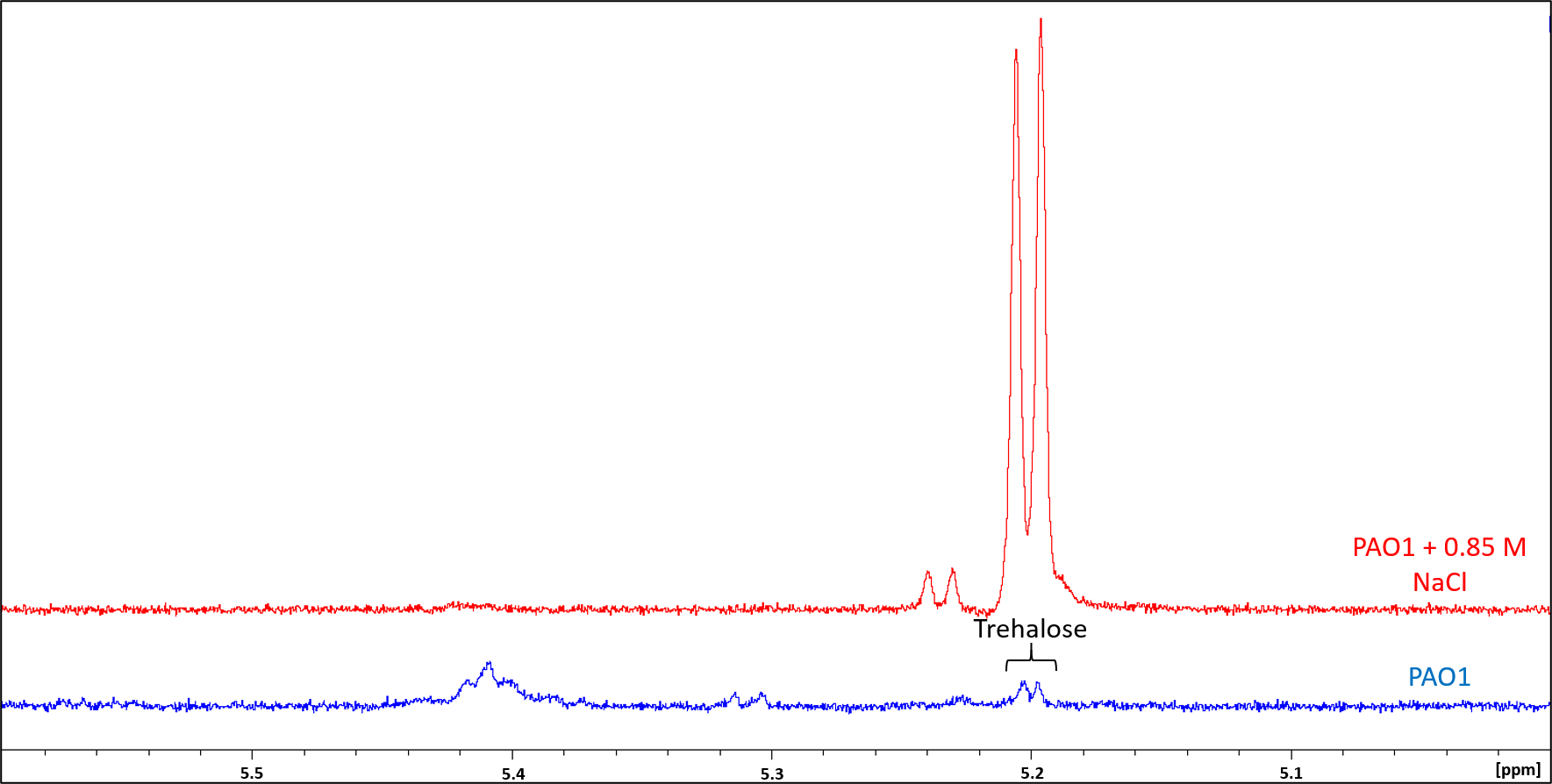

Supplement: S3 Fig — The peak corresponding to trehalose is indicated. (TIF) [file pgen.1009524.s003.tif]
