# Supplementary material for: Trehalose and α-glucan mediate distinct abiotic stress responses in Pseudomonas aeruginosa
Source: PLoS Genet. 2021 Apr 19;17(4):e1009524. doi: 10.1371/journal.pgen.1009524 (PMC8084333; doi:10.1371/journal.pgen.1009524)
Supplement: S1 Table — (DOCX) [file pgen.1009524.s004.docx]

**S1 Table. Strains and plasmids used in this study**

| Strain | Details | Source |
| --- | --- | --- |
| *P. aeruginosa* |  |  |
| PAO1 | Wild-type *Pseudomonas aeruginosa* PAO1 | [1] |
| PAO1 Δ*glgE* | Non-polar deletion of *PA2151* | This study |
| PAO1 Δ*treS*/*pep2* | Non-polar deletion of *PA2152* | This study |
| PAO1 Δ*glgB* | Non-polar deletion of *PA2153* | This study |
| PAO1 Δ*glgA* | Non-polar deletion of *PA2165* | This study |
| PAO1 Δ*treZ* | Non-polar deletion of *PA2164* | This study |
| PAO1 Δ*malQ* | Non-polar deletion of *PA2163* | This study |
| PAO1 Δ*treY* | Non-polar deletion of *PA2162* | This study |
| PAO1 Δ*glgX* | Non-polar deletion of *PA2160* | This study |
| PAO1 Δ*glgP* | Non-polar deletion of *PA2144* | This study |
| PAO1 Δ*glgA/E* | Non-polar deletion of *PA2165 & PA2151* | This study |
| PAO1 Δ*4* | Non-polar deletion of *PA2162 & PA2151-PA2153* | This study |
| PAO1 :: *otsA*/*otsB* | PAO1 containing *p_tac_*-*otsA*/*B* at the *att::*Tn*7* insertion site, Gm^R^ | This study |
| PAO1 Δ*treS*/*pep2* :: *otsA*/*otsB* | PAO1 Δ*treS*/*pep2* containing *p_tac_*-*otsA*/*B* at the *att::*Tn*7* insertion site, Gm^R^ | This study |
| PAO1 Δ*glgA* :: *otsA*/*otsB* | PAO1 Δ*glgA* containing *p_tac_*-*otsA*/*B* at the *att::*Tn*7* insertion site, Gm^R^ | This study |
| PAO1 Δ*glgA/E* :: *otsA*/*otsB* | PAO1 Δ*glgA/E* containing *p_tac_*-*otsA*/*B* at the *att::*Tn*7* insertion site, Gm^R^ | This study |
| *E. coli* |  |  |
| BL21-(DE3) pLysS | Sm^R^, K12 *recF143 lacI^q^ lacZΔ.M15*, *xylA,* pLysS | Novagen |
| DH5α | *endA*1, *hsdR*17(r_K_-m_K_+), *supE*44, *recA*1, *gyrA* (Nal^r^), *relA*1, Δ(*lacIZYA-argF*) U169, *deoR*, Φ80*dlacΔ(lacZ)M15* | [2] |
| Plasmids |  |  |
| pTS1 | Tet^R^, suicide vector; *ColE1*-replicon, *IncP-1, Mob, lacZ* | [3] |
| pTS1-*glg/tre* vectors | pTS1 with Δ*glg/*Δ*tre* constructs as *Bam*HI-*Hind*III inserts | This study |
| TOPO101 | Amp^R^, pET101 directional TOPO vector, N-term His_6_-tag | Invitrogen |
| pET21a(+) | Amp^R^, purification vector, C-terminal His_6_-tag | Novagen |
| TOPO101-*glg/tre* vectors | TOPO101 with *glg/tre* genes inserted | This study |
| pET21a-*treS/pep2* | pET21a with *treS/pep2* gene as *Nde*I-*BamH*I insert | This study |
| pME3087 | Tet^R^, suicide vector; ColE1-replicon, IncP-1, Mob | [4] |
| pME3087-*treS/pep2* | pME3087 with *treS/pep2* as an *Eco*RI*-Bam*HI insert | This study |
| pME6032 | Tet^R^, P_K_, 9.8 kb pVS1 derived shuttle vector | [5] |
| pME-*glgB* | pME6032 with PAO1 *glgB* as an *Eco*RI*-Xho*I insert | This study |
| pUC18T-mini-Tn*7*T-Gm | Amp^R^, Gm^R^, Tn*7* insertion vector | [6] |
| pUC18-mini-Tn*7*TGm-*otsA/B* | pUC18-miniTn*7*Gm with *p_tac_*-*otsA*/*B* fragment as a *Hind*III -*Spe*I insert. | This study |
| pTNS2 | Helper plasmid for *att::*Tn*7* insertion | [6] |

**References**

1. Holloway, B.W., *Genetic recombination in Pseudomonas aeruginosa.* Journal of General Microbiology, 1955. **13**(3): p. 572-81.

2. Woodcock, D.M., et al., *Quantitative evaluation of Escherichia coli host strains for tolerance to cytosine methylation in plasmid and phage recombinants.* Nucleic Acids Res, 1989. **17**(9): p. 3469-78.

3. Scott, T.A., et al., *An L-threonine transaldolase is required for L-threo-beta-hydroxy-alpha-amino acid assembly during obafluorin biosynthesis.* Nature Communications, 2017. **8**.

4. Voisard C, et al., *Biocontrol of root diseases by Pseudomonas fluorescens CHA0: current concepts and experimental approaches.* O'Gara F, Dowling DN, Boesten B (eds). Molecular Ecology of Rhizosphere Microorganisms. , 1994: p. 67-89.

5. Heeb, S., et al., *Small, stable shuttle vectors based on the minimal pVS1 replicon for use in gram-negative, plant-associated bacteria.* Mol Plant Microbe Interact, 2000. **13**(2): p. 232-7.

6. Choi, K.H., et al., *A Tn7-based broad-range bacterial cloning and expression system.* Nat Methods, 2005. **2**(6): p. 443-8.
