# Supplementary material for: Trehalose and α-glucan mediate distinct abiotic stress responses in Pseudomonas aeruginosa
Source: PLoS Genet. 2021 Apr 19;17(4):e1009524. doi: 10.1371/journal.pgen.1009524 (PMC8084333; doi:10.1371/journal.pgen.1009524)
Supplement: S2 Table — (DOCX) [file pgen.1009524.s005.docx]

**S2 Table. Primers used in this study**

| Target | No. | Primer Name | Primer Sequence (5′ – 3′) |
| --- | --- | --- | --- |
| *glgE* (*PA2151*) | 1 | PA2151_UPFP | CGCGGGATCCTGGACGAAAGCGTCAC |
|  | 2 | PA2151_UPRP | CGCGTCTAGAGACAATCGAACTCATCGAG |
|  | 3 | PA2151_DNFP | CGCGTCTAGAGTCGACGCATAAGGAGAAAC |
|  | 4 | PA2151_DNRP | CGCGAAGCTTAATAGAAGCGGTGCCAGTAG |
|  | 5 | PA2151_TestF | TGGTGCTGATCACCCTG |
|  | 6 | PA2151_TestR | GTTCGATCAGGTAGGGAATC |
| *treS*/*pep2* (*PA2152*) | 7 | PA2152_UPFP | CGGGATCCGACTGCTACCGGCCGAAC |
|  | 8 | PA2152_UPRP | CGTCTAGACGGTTTCTCGCGTCTGG |
|  | 9 | PA2152_DNFP | CGTCTAGACTCCACCGAACACCTCCTG |
|  | 10 | PA2152_DNRP | CGGAATTCCTCGTACTTGTACAGTTC |
|  | 11 | PA2152_TestF | CCGAACTCAACCAGCCAC |
|  | 12 | PA2152_TestR | CGGCAGGAGGCCGTCGTG |
| *glgB* (*PA2153*) | 13 | PA2153_UPFP | CGCGGGATCCACGCTTCATCGACTTCGAG |
|  | 14 | PA2153_UPRP | CGCGTCTAGAGAGGAAGGCGAATGGATC |
|  | 15 | PA2153_DNFP | CGCGTCTAGAGACGTGCTGCTCAATAGC |
|  | 16 | PA2153_DNRP | CGCGAAGCTTAGCAACTGGTCGCTGATG |
|  | 17 | PA2153_TestF | CTGCTGATCAGGGTTCAC |
|  | 18 | PA2153_TestR | GCCCTGCAAGGCCTGCTC |
| *glgA* (*PA2165*) | 19 | PA2165_UPFP | CGCGGGATCCATTACGCAAGGTCACCAG |
|  | 20 | PA2165_UPRP | CGCGTCTAGAAACAGAATGTCCCATGTC |
|  | 21 | PA2165_DNFP | CGCGTCTAGACGCCTGTTGAGGAATAC |
|  | 22 | PA2165_DNRP | CGCGAAGCTTCAGATGCTTGAGTTGCTC |
|  | 23 | PA2165_TestF | AGCACCTGGTTCAGCAACAG |
|  | 24 | PA2165_TestR | GAAGTGGTTGTAGATCACG |
| *treZ* (*PA2164*) | 25 | PA2164_UPFP | CGCGGGATCCTGGAGATCAGCGATGC |
|  | 26 | PA2164_UPRP | CGCGTCTAGATTGGAACTGGGCGCCGAAG |
|  | 27 | PA2164_DNFP | CGCGTCTAGAATCGCGGTCAGCCTG |
|  | 28 | PA2164_DNRP | CGCGAAGCTTCAGCAGGTCCTCGAGG |
|  | 29 | PA2164_TestF | AACACGCGCTACGTGGAG |
|  | 30 | PA2164_TestR | GCAGGACATTGAAGAACAGC |
| *malQ* (*PA2163*) | 31 | PA2163_UPFP | CGCGGGATCCAACCGTTCCTGTATTTCAC |
|  | 32 | PA2163_UPRP | CGCGTCTAGACAGGCGTGCGTCACTCATG |
|  | 33 | PA2163_DNFP | CGCGTCTAGATGCATCGGCTATGTCGG |
|  | 34 | PA2163_DNRP | CGCGAAGCTTTCCTGCCACCAGGGATTG |
|  | 35 | PA2163_TestF | GATAGCGCTGCAACTGCTC |
|  | 36 | PA2163_TestR | GGATTCCCACTGAATG |
| *treY* (*PA2162*) | 37 | PA2162_UPFP | CGCGGGATCCTGCTGCTCTTCGAG |
|  | 38 | PA2162_UPRP | CGCGTCTAGAGAAATAGTCCAGCCAG |
|  | 39 | PA2162_DNFP | CGCGTCTAGAGACTTTCCCGTCAACCTG |
|  | 40 | PA2162_DNRP | CGCGAAGCTTAACAGGCACAGCTCGACCTTG |
|  | 41 | PA2162_TestF | AACGTCTCTGGCTGATCC |
|  | 42 | PA2162_TestR | GTGTACTCGGGCAATTC |
| *glgX* (*PA2160*) | 43 | PA2160_UPFP | CGCGGGATCCAGTCTCTCCGAATGCAC |
|  | 44 | PA2160_UPRP | CGCGTCTAGATTCGCTGATCCGCGAAG |
|  | 45 | PA2160_DNFP | CGCGTCTAGAAGTTTCCTGCTGTTCG |
|  | 46 | PA2160_DNRP | CGCGAAGCTTAAGAAAGGCTCAGTAGTG |
|  | 47 | PA2160_TestF | CAGTCGTCACGGTTGCCTG |
|  | 48 | PA2160_TestR | ACGGCTTTCATGGCGTTC |
| *glgP* (*PA2144*) | 49 | PA2144_UPFP | CGCGGGATCCTTGGCAACGGCATTC |
|  | 50 | PA2144_UPRP | CGCGTCTAGAGAGGATGCTGGCTTTCAG |
|  | 51 | PA2144_DNFP | CGCGTCTAGAACGATCAGCGAATACG |
|  | 52 | PA2144_DNRP | CGCGAAGCTTGAATACCGAAGAATGC |
|  | 53 | PA2144_TestF | ATACGGGAAAGGTCACTG |
|  | 54 | PA2144_TestR | CCATGAATGGAATTTCGAACC |
| *M. koreensis* *3J1* *otsA/otsB* | 55 | OtsA/B FP | CGCGGAATTCCACCCATGCCAGCCGCA |
|  | 56 | OtsA/B RP | CGCGAGATCTGGACATGACGAGAGTCTATTCCCG |
| pME6032 *tac* cassette | 57 | ptacFP | CGCGAAGCTTGACATCATAACGGTTCTG |
|  | 58 | ptacRP | CGCGACTAGTGTCCGAGGAGCTTTATGC |
| *glgA* (*PA2165*) | 59 | PA2165_PurF | CACCATGGGACATTCTGTTGCAGGCGTTTG |
|  | 60 | PA2165_PurR | CTGGCGTACCCCGCGCAGG |
| *glgP* (*PA2144*) | 61 | PA2144_PurF | CACCATGCCCGACAGCCAAGACCAGACC |
|  | 62 | PA2144_PurR | TAGCTTCCAGATATCCCGGGCGTATTCGCTGATCGT |
| *glgE* (*PA2151*) | 63 | PA2151_PurF | CACCATGAGTTCGATTGTCCGCAACAGCG |
|  | 64 | PA2151_PurR | TGCGTCGACGCGCCGCAG |
| *malQ* (*PA2163*) | 65 | PA2163_PurF | CACCATGAGTGACGCACGCCTGGCCGAACTG |
|  | 66 | PA2163_PurR | ATCATGGGCGGCCTCCTCGGCATC |
| *glgB* (*PA2153*) | 67 | PA2153_PurF | CACCATGAGCGAACCGATGTCCGAACGCGAACG |
|  | 68 | PA2153_PurR | GCCGGCCGGACGCAGC |
|  | 69 | 2153_EcoRIFor | GTCAGCGAATTCATGAGCGAACCGATGTC |
|  | 70 | 2153_XhoIRev | CAGTCGCTCGAGTCAGCCGGCCGGACGCA |
| *glgX* (*PA2160*) | 71 | PA2160_PurF | CACCATGAGCAGAAAGCGTAGCCCCGCTCCTTCG |
|  | 72 | PA2160_PurR | TCCTTCCGCTCCGGCGCCC |
