# Supplementary material for: Trehalose and α-glucan mediate distinct abiotic stress responses in Pseudomonas aeruginosa
Source: PLoS Genet. 2021 Apr 19;17(4):e1009524. doi: 10.1371/journal.pgen.1009524 (PMC8084333; doi:10.1371/journal.pgen.1009524)
Supplement: S3 Table — Predicted means and standard errors of log10(CFU/ml) for desiccation assays in Figs 9 and 10, calculated by linear mixed modelling (see text for details). The response to desiccation is the difference between log10(CFU) at 100% and 75% RH. P-values were calculated by Z-tests using the normal distribution. (DOCX) [file pgen.1009524.s006.docx]

**S3 Table. Statistical significance between the desiccation responses of PAO1 and mutant strains**

Predicted means and standard errors of log_10_(CFU/ml) for desiccation assays in Figs 9 and 10, calculated by linear mixed modelling (see text for details). The response to desiccation is the difference between log_10_(CFU) at 100% and 75% RH. P-values were calculated by Z-tests using the normal distribution.

| **Mean log_10_(CFU/ml)** | **Condition** | | | **Difference** |
| --- | --- | --- | --- | --- |
| **Strain** | **100%** | **75%** | **T_0_** | **100% − 75%** |
| WT PAO1 | 7.6 | 6.5 | 6.3 | 1.1 |
| PAO1 :: *otsA/B* | 7.9 | 5.7 | 6.5 | 2.2 |
| Δ*alg* | 7.7 | 4.7 | 5.6 | 3.0 |
| Δ*glgA* | 7.4 | 4.8 | 6.2 | 2.6 |
| Δ*glgA* :: *otsA/B* | 7.7 | 6.9 | 6.4 | 0.9 |
| Δ*glgA ΔglgE* :: *otsA/B* | 7.6 | 4.4 | 6.2 | 3.2 |
| Δ*glgB* | 7.6 | 7.3 | 6.2 | 0.3 |
| Δ*treS/pep2* | 7.5 | 4.4 | 6.2 | 3.1 |
| Δ*treS/pep2* :: *otsA/B* | 7.7 | 3.7 | 6.4 | 3.9 |
|  |  |  |  |  |
| **Standard error of log_10_(CFU/ml)** | **Condition** | | | **SE of response** |
| **Strain** | **100%** | **75%** | **T_0_** | **100% − 75%** |
| WT PAO1 | 0.1 | 0.1 | 0.1 | 0.1 |
| PAO1 :: *otsA/B* | 0.3 | 0.3 | 0.3 | 0.4 |
| Δ*alg* | 0.3 | 0.3 | 0.3 | 0.5 |
| Δ*glgA* | 0.2 | 0.2 | 0.2 | 0.3 |
| Δ*glgA* :: *otsA/B* | 0.2 | 0.2 | 0.2 | 0.2 |
| Δ*glgA* Δ*glgE* :: *otsA/B* | 0.3 | 0.3 | 0.3 | 0.4 |
| Δ*glgB* | 0.3 | 0.3 | 0.3 | 0.4 |
| Δ*treS/pep2* | 0.2 | 0.2 | 0.2 | 0.2 |
| Δ*treS/pep2* :: *otsA/B* | 0.2 | 0.2 | 0.2 | 0.3 |
|  |  |  |  |  |
| **Difference from WT PAO1 in desiccation response** | | | | |
|  | **Difference of** | **SE of** |  |  |
| **Strain** | **responses** | **difference** | **P value** |  |
| PAO1 :: *otsA/B* | 1.1 | 0.4 | 0.0044 | ** |
| Δ*alg* | 1.9 | 0.5 | <0.0001 | **** |
| Δ*glgA* | 1.5 | 0.3 | <0.0001 | **** |
| Δ*glgA* :: *otsA/B* | -0.2 | 0.3 | 0.35 | ns |
| Δ*glgA* Δ*glgE* :: *otsA/B* | 2.1 | 0.4 | <0.0001 | **** |
| Δ*glgB* | -0.8 | 0.4 | 0.036 | * |
| Δ*treS/pep2* | 2.0 | 0.3 | <0.0001 | **** |
| *ΔtreS/pep2* :: *otsA/B* | 2.8 | 0.3 | <0.0001 | **** |
