# Supplementary material for: Trehalose and α-glucan mediate distinct abiotic stress responses in Pseudomonas aeruginosa
Source: PLoS Genet. 2021 Apr 19;17(4):e1009524. doi: 10.1371/journal.pgen.1009524 (PMC8084333; doi:10.1371/journal.pgen.1009524)
Supplement: Attachment — Submitted filename: 111651_1_rebuttal_2027367_qqbvk0.pdf [file pgen.1009524.s007.pdf]

Response to reviewers: Woodcock et al. 2021

REVIEW COMMONS

Reviewer 1 (Evidence, reproducibility, and clarity):

Summary

The authors resolved the biosynthesis of trehalose and alpha-glucan in *Pseudomonas aeruginosa* and the role of these two compounds in osmotic and desiccation stress.

We thank the reviewer for their positive review of our manuscript. Our responses to their specific queries are interspersed below.

Major comments:

• *Are the key conclusions convincing?*

Yes

• *Should the authors qualify some of their claims as preliminary or speculative, or remove them altogether?*

No

• *Would additional experiments be essential to support the claims of the paper? Request additional experiments only where necessary for the paper as it is, and do not ask authors to open new lines of experimentation.*

Not necessary, comprehensive coverage of research topic.

• *Are the suggested experiments realistic in terms of time and resources? It would help if you could add an estimated cost and time investment for substantial experiments.*

Not applicable

• *Are the data and the methods presented in such a way that they can be reproduced?*

Yes

• *Are the experiments adequately replicated and statistical analysis adequate?*

Yes, everything is adequate but just one subtle concern: check the significance of the number of digits in the entries listed in Table S3. Revise Table S3.

Table S3 has been revised as requested. The data in this table is now presented correct to one decimal place.

Minor comments:

• *Specific experimental issues that are easily addressable.*

Not applicable (Table S3: see above)

• *Are prior studies referenced appropriately?*

No. Refs. 18- 32: The subjects of 'trehalose' and 'osmotic stress' have already been addressed in the *Pseudomonas* field and should be referenced. The authors cite work carried out on trehalose and osmotic stress on phylogenetically distant microorganisms, but do not cite related work from the *Pseudomonas* field which I consider to be inappropriate. Similarly, trehalose biosynthesis in *Pseudomonas* has not only been covered by refs. 47 and 48.

This is a fair comment. The focus of our introduction came from a desire to concentrate specifically on the metabolism and intracellular function of trehalose/α-glucan in *Pseudomonas*. In hindsight, we acknowledge that our introduction is a little too narrowly focussed. We have expanded the introduction and discussion sections to include additional discussion of trehalose in *Pseudomonas* and its regulation in the CF lung.

• *Are the text and figures clear and accurate?*

Extremely well written manuscript and prepared figures

• *Do you have suggestions that would help the authors improve the presentation of their data and conclusions?*

Revise the list of references and discuss more thoroughly your novel findings in the light of existing knowledge in the *Pseudomonas* field.

Please see previous comment relating to the literature.

Reviewer 1 (Significance):
Significance
• Describe the nature and significance of the advance (e.g. conceptual, technical, clinical) for the field. Conceptual advance: The authors identified and characterized the enzymatic pathway of trehalose and alpha-glucan biosynthesis in *Pseudomonas aeruginosa* and its role to cope with osmotic and desiccation stress. The authors' conclusions do not correspond with recently published peers' work; hence they should discuss in more detail why they consider their data to be more accurate to discern the role of trehalose to contain desiccation and osmotic stress in *P. aeruginosa*.
Please see previous comment relating to the literature. In general, the published work to date on trehalose in *Pseudomonas* spp. does not consider GlgE pathway-mediated link to α-glucan that we characterise in this paper. Our work demonstrates that synthesis and metabolism of the two molecules are implicitly linked in species where the GlgE pathway is present, and they cannot be considered in isolation. For this reason we are very confident that our study represents the most accurate model to date for trehalose and α-glucan metabolism and their associated phenotypes in *P. aeruginosa*. We have therefore emphasised that the role of trehalose in *Pseudomonas* spp. should be re-evaluated in light of our findings.

• Place the work in the context of the existing literature (provide references, where appropriate). Existing literature focusing on trehalose, osmotic stress, desiccation stress in the *Pseudomonas* field not cited by the authors:
>>>
These papers are of variable scientific quality, but the conceptual work by Hallsworth and the work by Behrens on the PA metabolome in CF lungs are worth discussing. All other work provides pieces of information on function and biosynthesis of trehalose up to now known by the *Pseudomonas* community. The authors resolved the function of the GlgA operon which will be definitely appreciated.
We thank the reviewer for these helpful suggestions. We have reviewed these papers carefully and have incorporated several, including the papers from Hallsworth and Behrens into the revised manuscript.

Strengths of the manuscript:

• Meticulously planned and carefully executed experiments, not a single experimental flaw

• Very high technical quality of experiments and primary data

• Comprehensive coverage of the research topic

• Excellent presentation in text and illustrations

Only weakness:
• Insufficient consideration of peers' published work on trehalose and its role in stress response in *P. aeruginosa*
Please see previous comment relating to the literature.

• State what audience might be interested in and influenced by the reported findings. Scientists working in the fields of glycoconjugate and carbohydrate research, biochemists, microbiologists with interest in metabolic pathways, stress response and/or *Pseudomonas*.

Reviewer #2 (Evidence, reproducibility, and clarity):

It will be difficult for me to write a review of this paper and for the authors to make sense of my review because the manuscript's pages / lines are not numbered…

We apologise to the reviewer for this oversight.

Summary

The authors carried out a comprehensive characterization of the metabolism of trehalose in Pseudomonas aeruginosa PA01, using techniques of biochemistry, reverse genetics, and bioinformatics. The main findings include that the disaccharide trehalose is synthesized in this organism from branched chain α-glucans and that the catabolism of trehalose proceeds via another disaccharide, maltose and is fed back into the synthesis of α-glucans. Trehalose and α-glucans have been implicated in conferring resistance to abiotic stresses in other organisms. The authors show that mutants that are blocked in the synthesis of trehalose are sensitive to high salinity but are normal with respect to their sensitivity to desiccation, whereas mutants impaired in the accumulation of α-glucans are sensitive to desiccation without being unduly sensitive to osmotic stress. These results indicate that trehalose and α-glucans have different roles in abiotic stress-tolerance.

Major points

This manuscript describes an impressive amount of careful work and presents new insights into the metabolism of trehalose, maltose, and α-glucans. However, the authors should address the following major comments before the paper is accepted.

We thank the reviewer for their thorough and positive assessment of the manuscript. We address their specific points below.

1. Discussion: the authors state that "trehalose protects *Pseudomonas* ssp. against osmotic stress, most likely due to its role as a compatible solute." According to Table 2, *P. aeruginosa* grown in the medium of low osmolarity accumulated 0.13% trehalose per gram dry weight, i.e. ~4 µmol / g dry weight. Assuming that the dry weight / wet weight ratio of *P. aeruginosa* is the same as that of *P. putida*, which is ~1/3 (PMID: 6508285), the concentration of trehalose in the cells calculates to be ~2 mM. It is not plausible that trehalose could be significant as compatible solute at this low concentration.

One way out could be if the accumulation of this disaccharide were increased by osmotic stress. The authors should also measure the trehalose content of cells grown in medium containing 0.85 M NaCl. In case of positive results in this experiment, it would be interesting to determine the effects of osmotic stress on the levels of trehalose biosynthetic and catabolic enzymes, but this would not be necessary for the acceptance of the paper.

This is a fair point. To address this, we measured the trehalose and maltose-1-phosphate levels for PA01 grown in the presence of 0.85 M NaCl. We saw a highly significant increase in the abundance of trehalose, compared to growth on standard M9 media. This strongly suggests that trehalose accumulates under conditions of osmotic stress as suggested by the reviewer. These new results have been added to the relevant sections of the manuscript (M&M, results, table 2 and discussion). The student (Danny Ward) who conducted these new experiments has been added to the author list.

However, there is also an extensive literature suggesting that trehalose has antioxidant functions e.g. PMID: 29241092 (the first paper that came up in Google search for "trehalose as antioxidant"). The authors should discuss this possible alternate role of trehalose.

The reviewer is correct that trehalose has well-documented antioxidant functions in various species. We have modified the introduction to address this. To maintain the focus of our manuscript on bacteria we have used a different example to that suggested by the reviewer.

It is not described adequately in the Materials and Methods how the cellular contents of trehalose and maltose-1-phosphate (M1P) were determined.

The Materials and Methods section has been revised to include more details of this method.

2. I found the growth curves in Figure 8, especially in panel B, to be uninterpretable. The authors should spread these data into more panels or use some other method to make them clearer.

We have expanded the legend for Figure 8 to describe more fully what is going on in this figure. The results in Figure 8 are grouped according to the operons in which each set of genes is located. As such, the graphs contain unequal numbers of curves, with 8B containing the most and 8C only showing data for WT and Δ*glgP*.

3. The statement "The GlgA and GlgE proteins . . . enable two alternate mechanisms for linear α-glucan biosynthesis", which is echoed a number of times in the manuscript, seems to create the impression that there are two de novo pathways of synthesis of these polysaccharides. However, as shown in Figure 1, the GlgA pathway is the only route to the net synthesis of α-glucans, and GlgE is only part of a recycling pathway. Therefore, it cannot be true that "the vast majority of α-glucan accumulated by *P. aeruginosa* will be produced by GlgE".
We have revised this section to further clarify what we mean when we state that the majority of α-glucan accumulated by *P. aeruginosa* will be produced by GlgE. Our data suggest that there is a big difference between the *generation* of α-glucan (conducted by both GlgA and GlgE) and its *accumulation* (flux through GlgA generated α-glucan is high, so only GlgE generated α-glucan can accumulate to generate large polymers).

4. The authors state that "MalQ disproportionates (sic) α-glucan with glucose to produce maltose." Figure 1 shows that GlgE uses an "acceptor", which I assume could be glucose. How is free glucose synthesized? Could cells grown on a non-carbohydrate as sole carbon source make free glucose?
P. aeruginosa is able to carry out gluconeogenesis, so it can produce glucose from non-carbohydrate carbon sources if necessary.

Our data show that GlgE acceptor preference gets lower as the acceptor molecule gets shorter. It is possible to detect GlgE activity without an acceptor. In this case we see a lag, implying M1P hydrolyses slowly at first and priming with glucose is also slow. Eventually however, the products get long enough for the reaction to take off. MalQ will work with DP2 or longer as the donor and DP1 or longer as the acceptor, moving one glucose unit at a time.

Pedantic point, but "disproportionation" means an oxidation-reduction reaction in which two identical molecules are used to produce two different molecules (https://en.wikipedia.org/wiki/Disproportionation). The reaction catalysed by MalQ does not involve electron transfer. Don't the authors mean that this enzyme is a glycosyl transferase?
We have checked this, and our use of disproportionation in the manuscript is correct. The definition of disproportionation is any desymmetrizing reaction of the following type: 2 A → A' + A", and is not limited to redox reactions. MalQ carries out a reaction of this type when presented with a maltooligosaccharide.

5. The authors state that TreS had "a very high Km for trehalose (>100 mM)". In view of the low concentration of trehalose (Point 1, above), the physiological relevance of this suggested activity is questionable. See response to question 1 above. As trehalose levels are elevated under osmostress conditions this concern becomes less critical. It is of course true that conditions *in vitro* may not fully reflect cellular conditions and that this activity may be higher *in vivo*, but this is a general limitation of all protein biochemistry studies. The important point here is that trehalose synthase activity is detected for PA01 TreS.

6. Explain better what "predicted mean log10(CFU) means.
The predicted mean refers to the value of log10(CFU) predicted by the statistical model we use. We have clarified this in the relevant sections of the manuscript.

7. Can the authors suggest how "α-glucan protects PA01 against desiccation"?
Without further investigation we can only speculate as to how α-glucan confers desiccation tolerance in PA01. One possibility is that α-glucan functions as a hydrogel, like the exopolysaccharide alginate, trapping water molecules and slowing their evaporation. Alternatively, it may confer a structural role akin to that of trehalose, preventing the loss of cell integrity as water levels decrease. We now address these possibilities in the discussion.

8. Can *P. aeruginosa* metabolize exogenous trehalose or maltose? If the authors know either way, they should mention it. If they don't know, I am not suggesting that they should test this for this paper, but it would be interesting to know whether these compounds would induce the expression of the trehalose or maltose catabolic enzymes or repress the relevant biosynthetic enzymes.
P. aeruginosa is able to metabolise exogenous maltose and trehalose. While the experiments that the reviewer suggests are certainly interesting, in our view *tre/glg* gene regulation is beyond the scope of the current manuscript. This field is certainly worth investigating in the future, however.

Minor points

9. First page under "Results": "phosphomutase" should be "phosphoglucomutase"?
Changed as requested.

10. Discussion: insert "*P. syringae*" before "*Pto*".
Changed as requested.

11. Materials and Methods: describe how ADP was quantified in the maltokinase assay.
The materials and methods section has been updated as requested.

Reviewer 2 (Significance):

Significance

Until this work, the biosynthesis of trehalose has been most extensively characterized in *Escherichia coli*, in which it has been shown that this disaccharide is made by the reaction of glucose-6-phosphate and UDP-glucose to give trehalose-6-phosphate and dephosphorylation to trehalose, catalysed by OtsA and OtsB. The authors discovered a very different pathway in *P. aeruginosa* in which the synthesis of trehalose goes through α-glucans as intermediates.

Because trehalose and α-glucans are needed for osmotic stress- and desiccation-tolerance, respectively, this work is of significance to researchers studying abiotic stress resistance.

PLOS PATHOGENS

Part I – Summary Please use this section to discuss strengths/weaknesses of study, novelty/significance, general execution, and scholarship.

Reviewer #1: The authors have presented data on the interconnectedness of trehalose and a-glucan in *Pseudomonas aeruginosa* and the roles for trehalose in osmostress response and not in desiccation, where a-glucan appears to be most important. The responses to previous reviews were appropriate and this is an important contribution to our understanding of P. aeruginosa survival, particularly on surfaces.

The only major weakness I note is that the authors conduct the study in media lacking the very common exogenous osmoprotectants and their precursors that P. aeruginosa can find and acquire from its environment: proline, choline (converted to GB for osmoprotection), carnitine, stachydrine, and proline betaine. I do not think this a critical issue, but something that must be appropriately noted as a caveat to the conclusion statements. For instance, in the abstract, the authors state that "trehalose is vital to the PAO1 osmotic stress response..." which is only true in the absence of exogenous osmoprotectants and their precursors.

We thank the reviewer for their positive comments on our study. To avoid potentially confusing the results of our experiments we carefully defined the experimental conditions and did not include additional sources of osmoprotectants beyond the proline present in the assay media (M9 + glucose + casamino acids). Nonetheless, we appreciate the reviewer's point and have qualified our conclusions in several places in the manuscript.

Reviewer #2: Woodcock et al. report a physiological study evaluating the biosynthesis and role of trehalose and alpha-glucan in desiccation and osmotic stress tolerance in P. aeruginosa by using reverse genetics and analytical biochemistry. The main findings are that trehalose protects Pseudomonas spp. against osmotic stress while alpha-glucan protects cells against desiccation stress. This work is original and quite interesting. However, in its current form it does provide any information if the pathways are involved in pathogenesis or not. Thus, it is a preliminary in this context. I have some comments the authors could consider to enhance the manuscript.

Our paper aims to define the specific roles of trehalose & α-glucan in *P. aeruginosa* osmoprotection & desiccation and the consequences of this for pathogen survival in the clinical/built environment. The possible contributions of these pathways to pathogenesis are intriguing, and we speculate on these in the discussion. However, this is beyond the scope of the research study presented here. The effort required to properly test this hypothesis would make a substantial manuscript in its own right.

Reviewer #3: In the submitted manuscript, Woodcock and colleagues dissect the functions of trehalose and alpha-glucan biosynthesis pathways in P. aeruginosa, using lab strain PAO1 as the model. I appreciated the targeted metabolomics of carbohydrates using NMR, which is not technically trivial. In vivo studies done by metabolomics were corroborated with data from in vitro protein biochemistry. Phenotypic profiling of bacterial mutants provides evidence for physiological relevance to osmotic/desiccation stress tolerance.

We thank the reviewer for their positive assessment.

Part II – Major Issues: Key Experiments Required for Acceptance Please use this section to detail the key new experiments or modifications of existing experiments that should be absolutely required to validate study conclusions. Generally, there should be no more than 3 such required experiments or major modifications for a "Major Revision" recommendation. If more than 3 experiments are necessary to validate the study conclusions, then you are encouraged to recommend "Reject".

Reviewer #1: No critical experiment needed

Reviewer #2: 1. The main limitation of this work is that there is no data provided on the role of trehalose and alpha-glucan biosynthetic genes in pathogenesis. An in vitro and/or in vovo experiments needs to be done by using the strains generated in the present study to address this. Without evidence on the role of genes in infection, the paper is more of environmental science than a pathogen/infection.

Please see comment in part I above. This is beyond the scope of the current manuscript.

2. Within the discussion very interesting hypotheses are mentioned and if any of these hypotheses could be tested that would highly increase the impact of the paper.

Please see comment in part I above. This is beyond the scope of the current manuscript.

3. The use of a linear mixed modelling for the data presented in Figure 9 is unjustifiable. As clearly stated in the M & M section, under 'Desiccation tolerance assays', the authors have determined the CFUs. So, why not presenting the recovered CFU directly in their Figure than using a model to predict CFU to present the already determined CFU data?
The predicted mean is simply the technical term for a mean calculated in an experiment in which there is some kind of structure, in this case a complicated set-up of biological and technical replicates. Presenting the arithmetic mean of CFU would be incorrect; depending on how much structure there is in the experiment, it could even be highly misleading. Linear mixed modelling is the standard method for analysing a structured experiment of this nature. We have modified the text in places to clarify why linear mixed modelling was used in this case.

Reviewer #3: The resolution of metabolic pathways leading to trehalose and alpha-glucan synthesis are significant contributions to understanding P. aeruginosa physiology. The authors' dissection of these pathways in contributing to different stress tolerance phenotypes (osmotic vs. desiccation) is novel. I like the experiment at the end showing contributions to survival on steel surfaces. This experiment seems physiologically relevant.

Part III – Minor Issues: Editorial and Data Presentation Modifications Please use this section for editorial suggestions as well as relatively minor modifications of existing data that would enhance clarity.

Reviewer #1: The authors need to carefully go through and convert all PA01 to PAO1 (use the letter O, not the number 0, as the letter) refers to the O-antigen of that strain). In some cases, they use both in the same table. All components of the manuscript need to be checked, including the abstract.
We apologise for this oversight, which has now been corrected throughout.

Reviewer #2: 1. Figure legends need to have a summary sentence followed by descriptions of each panels. Please adjust accordingly (Figure 1 and 2).
The figure legends have been revised in places for consistency.

2. Figure 2A – Please add the m/z value for DP4 consistent with the other DPs depicted.
The missing m/z value is 689.258, and has been added to Fig 2A.

3. I cannot find any information on how the strains were grown for up to 150 h (Figure 8). The authors need to provide a detailed information on how the strains were grown and how the OD600 measurements were performed.
Experiments were conducted with continuous incubation and OD600 measurement in a temperature controlled, 96 well plate reader. The methods section has been updated with this information.

4. Figure 8 - why is the X-axis labels different for all the three panels?
The X-axes in Figure 8 were chosen to display the results as clearly and unambiguously as possible. As there are more plotlines on 8B, we judged that displaying a longer time period would present the data more clearly.

5. There is no mention of data presented in Figure 8C in the result section.
A reference to Figure 8C has been added to the relevant results section.

6. Figure 9A, y-axis Log10. 10 is missing.
The figure axis has been corrected.

7. I cannot see the advantage of presenting Figure 11 in two panels and unlabelled. It is better to merge the two panels.
This experiment was quite labour intensive and involved a lot of dilution plating. To ensure accuracy we split the experiment into two parts, hence the two panels. We considered merging these panels after the fact, but this would require a degree of (legitimate) data manipulation to normalise all values to a single WT control. In the end we decided to present the data as-is, to minimise this step.

8. Table captions needs to be written on the top of table not at the bottom (Table 1 and 2).
We are happy to modify the manuscript to meet all journal style requirements post-review.

9. Supplementary Figure 2 – For the ease of reading, it is better to label with their corresponding strains rather than an alphabet based labelling (as in Figure 6A).
We have edited this figure to include strain details alongside the original labelling.

10. The authors need to provide a more detailed information on the MALDI-TOF mass spectrometry acquisition, processing, and analysis of mass spectrometry data.
A section on MALDI-TOF mass spectrometry has been added to the Materials and Methods.

Reviewer #3: Reading this manuscript was a pleasure. I see that it has been revised already in response to reviewer comments. I have very little that I can add, other than to say that it will make a nice contribution to our field.
We thank the reviewer for their positive assessment of our manuscript, and we’re glad they liked it!
